# Case Report of a Child after Hematopoietic Cell Transplantation with Acute* Aspergillus* Tracheobronchitis as a Cause for Respiratory Failure

**DOI:** 10.1155/2016/9676234

**Published:** 2016-11-09

**Authors:** Stefanie Gauguet, Kate Madden, Jennifer Wu, Christine Duncan, Gi Soo Lee, Tonya Miller, William C. Klingensmith, Sandra K. Burchett, Meredith van der Velden

**Affiliations:** ^1^Division of Pediatric Critical Care, Department of Pediatrics, UMass Memorial Children's Medical Center, University of Massachusetts Medical School, 55 North Lake Avenue, Worcester, MA 01655, USA; ^2^Division of Critical Care Medicine, Department of Anesthesiology, Perioperative and Pain Medicine, Boston Children's Hospital, Harvard Medical School, 300 Longwood Avenue, Boston, MA 02115, USA; ^3^Division of Hematology/Oncology, Boston Children's Hospital, Harvard Medical School, 300 Longwood Avenue, Boston, MA 02115, USA; ^4^Department of Otolaryngology and Communication Enhancement, Boston Children's Hospital, Harvard Medical School, 300 Longwood Avenue, Boston, MA 02115, USA; ^5^Department of Pathology, Midland Memorial Hospital, Midland, TX 79701, USA; ^6^Division of Infectious Diseases, Boston Children's Hospital, Harvard Medical School, 300 Longwood Avenue, Boston, MA 02115, USA

## Abstract

Rapid respiratory failure due to invasive mycosis of the airways is an uncommon presentation of* Aspergillus* infection, even in immunocompromised patients, and very few pediatric cases have been reported. Patients with* Aspergillus* tracheobronchitis present with nonspecific symptoms, and radiologic studies are often noninformative, leading to a delay in diagnosis. Prompt initiation of adequate antifungal therapies is of utmost importance to improve outcome. We report the case of a 9-year-old girl with chronic myelogenous leukemia who developed respiratory distress 41 days after hematopoietic cell transplantation and rapidly deteriorated despite multiple interventions and treatment modalities.

## 1. Introduction

*Aspergillus* tracheobronchitis (AT) is a rare but severe form of invasive pulmonary aspergillosis. AT is associated with very high mortality, especially in neutropenic patients (90%) [1], highly immunocompromised patients with hematologic malignancies, and patients undergoing hematopoietic cell transplantation (70%, reviewed in [2]). Patients generally present with nonspecific symptoms, such as cough, fever, and respiratory distress, and radiologic studies are often noninformative, in as many as 47% according to a recent review [1], which may lead to a delay in diagnosis.

Prompt initiation of adequate antifungal therapies is of utmost importance to improve outcome. Surgical removal of infected tissue and adjunctive therapies to help relieve airway obstruction should be considered early to improve survival rates. Direct bronchoscopy with visualization of the airways is the gold standard for diagnosis and is important for obtaining tissue for microscopic analysis and culture. However, this is often not performed without hesitation, as it is an invasive procedure and carries risks, such as potentially fatal hemorrhage [3].

Over 150 cases of AT have been described in the literature over the last few decades, but only very few pediatric patients have been reported to date [4, 5]. Thus we describe the case of a 9-year-old girl with chronic myelogenous leukemia status after hematopoietic cell transplantation (HCT) who developed severe pseudomembranous and obstructive AT, respiratory failure, and subsequent death despite multiple aggressive interventions.

## 2. Case Report

The patient was a 9-year-old girl with chronic myelogenous leukemia that presented in blast crisis who underwent 5/6 HLA-mismatched unrelated umbilical cord blood transplant following conditioning with total body irradiation, cyclophosphamide, and antithymocyte globulin. She had achieved cytogenetic and molecular remission prior to transplant, and neutrophil engraftment occurred on day 30. Before transplant, she had suffered two episodes of coagulase-negative staphylococcal bacteremia, but after the transplant she had an initially uncomplicated course without infectious issues. She received antibiotic prophylaxis with ampicillin-sulbactam empirically after transplant and cyclosporine and prednisone for graft versus host disease (GVH) prophylaxis. Two days prior to symptom development, the patient underwent elective percutaneous endoscopic gastrostomy tube placement. Thirty-nine days after transplant, she developed low-grade fevers, a sore throat, rhinorrhea, a nonproductive cough, and a purple-colored macular rash. Blood, urine, and nasopharyngeal samples were sent for cultures and viral testing, which remained negative, and empiric antibiotic coverage was broadened. Of note, surveillance cultures from the patient's nares and throat were sent on admission and weekly thereafter, which all remained negative. Rectal swab surveillance revealed vancomycin-resistant* Enterococcus faecium.*

**Figure 1:**
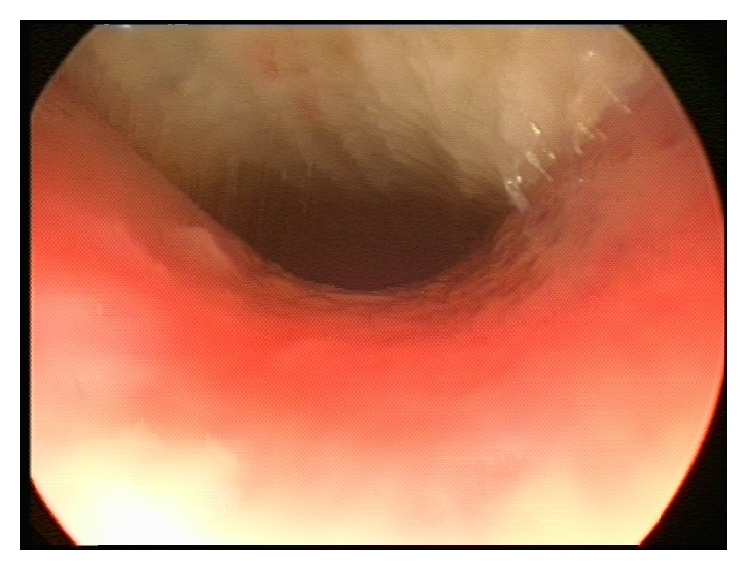
View of the trachea during bronchoscopy. Note the obstruction of >50% of the tracheal lumen by whitish thick secretions.

**Figure 2:**
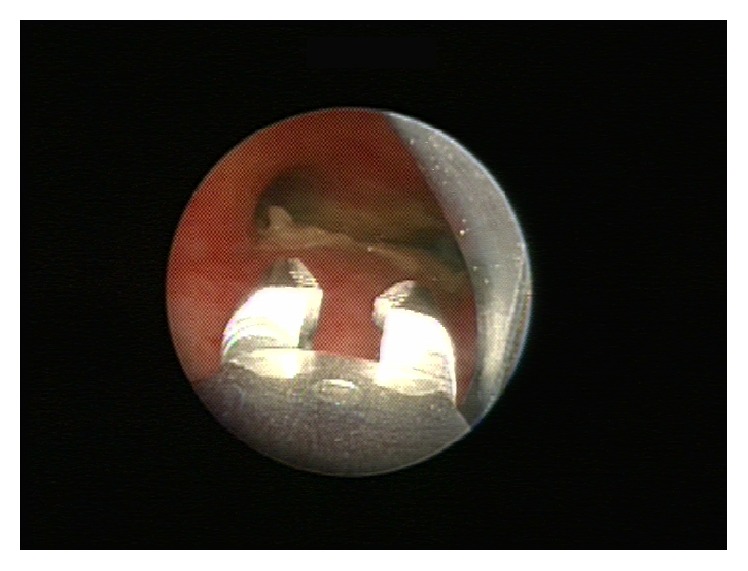
View of the carina during bronchoscopy. Note the thick, pseudomembranous white material obstructing a great portion of both mainstem bronchi and the optical foreign body forceps in place used to gently remove these.

Two days later, she developed mild respiratory distress with diffuse scattered wheezing bilaterally on exam. A CXR was unchanged and normal. With worsening respiratory course, antimicrobial therapy included IV clindamycin (12 mg/kg every eight hours), vancomycin (20 mg/kg every 6 hours), meropenem (20 mg/kg every 8 hours), ambisome (3 mg/kg daily with planned increase to 5 mg/kg when fungal elements were seen), azithromycin 5 mg/kg daily, and with further progression a single dose of cidofovir (5 mg/kg). With fungal elements obtained from tracheal tissue and with progressive* Aspergillus* infection in the differential diagnosis, posaconazole was added (enteral with formula at approximately 5-6 mg/kg given 4 times daily). The following day, she developed stridor and supra- and substernal retractions. A repeat CXR was again unchanged, but lateral neck films showed subglottic airway narrowing with soft tissue fullness of the glottis and subglottic areas. She was transferred to the pediatric intensive care unit for increasing respiratory distress. Noninvasive positive pressure ventilation in the form of BIPAP caused neither relief of her symptoms nor improvement of aeration. She was brought to the operating room for direct laryngoscopy and tracheobronchoscopy, which revealed an erythematous and edematous supraglottis and extensive pseudomembranous and obstructive tracheitis. An estimated 50–60% of the entire tracheal lumen was filled with thick, whitish secretions, in addition to a mucosal pseudomembrane that was visualized down to the carina (Figure 1). After mechanical removal of the secretions and pseudomembranes (Figure 2), she was left intubated and readmitted to the intensive care unit for further care.

Over the next few hours, she developed worsening biphasic airway obstruction.

She was increasingly difficult to ventilate despite several different attempted modes of ventilation and muscle relaxation. Treatment adjuncts included heliox (a mixture of helium and oxygen), to decrease airway turbulence, inhaled albuterol, intravenous terbutaline, and ketamine infusions for bronchodilation.

A repeat airway evaluation the following day revealed near-obstructive tenacious material adherent to the mucosa of the entire trachea. Thick mucoid secretions obscured all visible bronchi. Again, operative debridement of the pseudomembranes was attempted as distally as possible, but without significant improvement in her status. Trials of nebulized dornase alfa, bicarbonate, and acetylcysteine did not ameliorate the airway obstruction.

**Figure 3:**
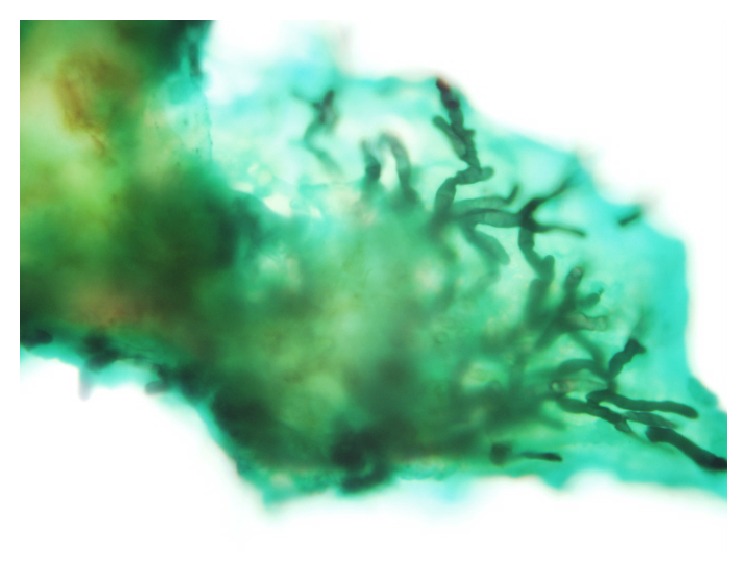
Grocott's methenamine silver stain of a piece of pseudomembrane obtained during bronchoalveolar lavage showed dichotomously branched and septate hyphae, suggestive of the diagnosis of* Aspergillus*. 400-fold magnification.

Microscopic examination of the debris obtained by bronchoalveolar lavage confirmed fungal elements consistent with* Aspergillus fumigatus *(Figure 3). Sputum and airway tissue cultures eventually grew* Aspergillus fumigatus *5 days postmortem as well. Systemic antifungal therapy was broadened to include nebulized amphotericin B (2.5 mg/hour) and enteral posaconazole (200 mg PG q6h 5-6 mg/kg 4 times daily).

In discussion with the patient's family, given their daughter's poor prognosis, the decision was made to withdraw life-supporting treatments. Mechanical ventilation was discontinued, comfort ensured with titration of opioids and benzodiazepines, and the patient passed away in the presence of her family.

**Figure 4:**
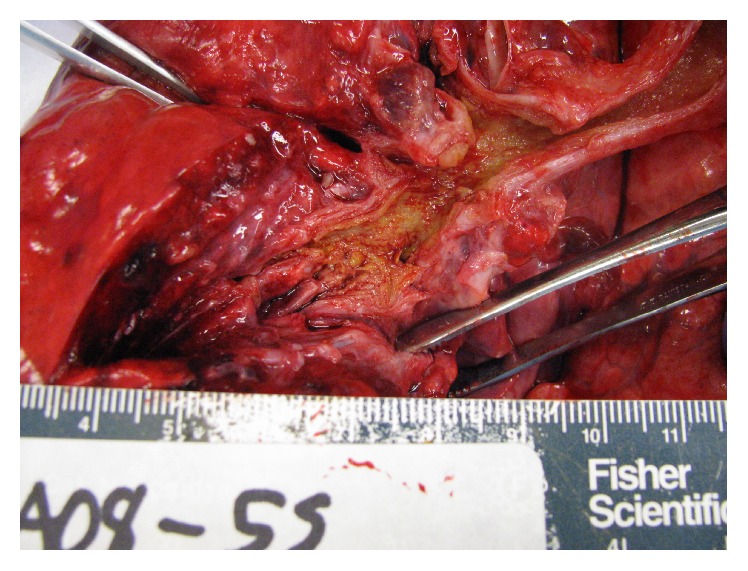
Autopsy: posterior view of left bronchial tree demonstrating the complete filling of even most distal airways with yellow gelatinous material.

**Figure 5:**
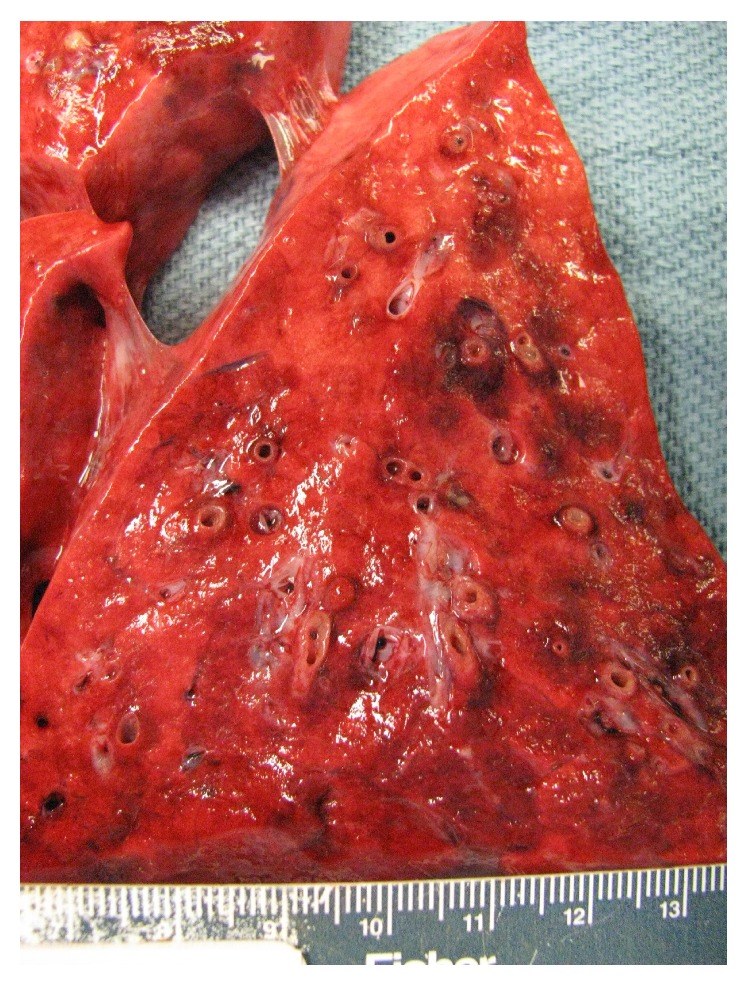
Autopsy: coronal cut through lung demonstrating peribronchial congestion and hemorrhage.

**Figure 6:**
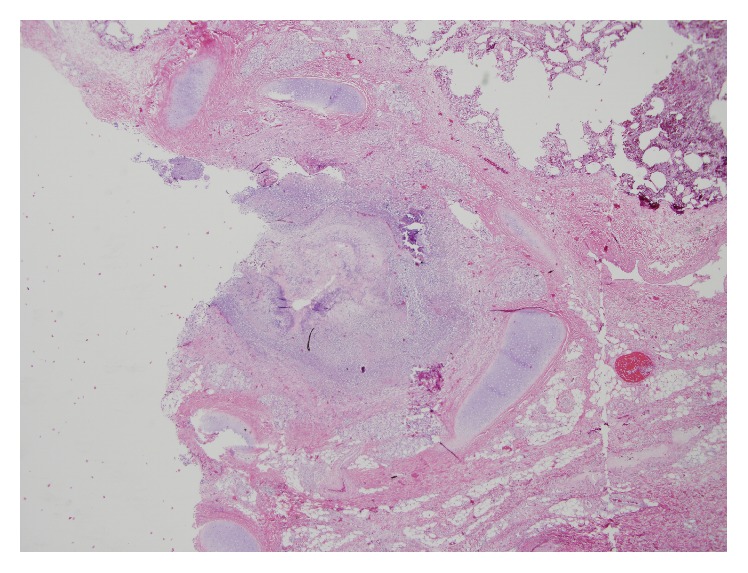
Histopathology: 10-fold magnification and H+E stain of a large bronchus demonstrating an entirely obstructed lumen.

Postmortem examination revealed tan-yellow, tenacious patchy tracheal membranes. The entire bronchial tree was lined with tan-yellow material and all lumen were narrowed or obstructed at the level of the small intrasegmental bronchi (Figures 4 and 5). There was peribronchial congestion and hemorrhage (Figure 6), as well as hilar and mediastinal lymphadenopathy. Autopsy cultures confirmed* Aspergillus fumigatus*.

## 3. Discussion

This case demonstrates an acute presentation of* Aspergillus* tracheobronchitis in a 9-year-old girl after HCT with nonspecific respiratory symptoms initially, including a nonproductive cough and sore throat, followed by scattered wheezes, and subsequently mild stridor. After this three-day prodrome, the patient developed rapidly progressive respiratory failure requiring multiple interventions without success in treating her infection and airway obstruction.

Although invasive aspergillosis is an uncommon diagnosis, immunosuppressed patients who present with fever and nonspecific respiratory symptoms, including airway obstruction and bronchospasm or even unilateral wheezing [6], should be evaluated for* Aspergillus* tracheitis promptly, ideally by bronchoscopy [2, 7]. Bronchoscopy has been reported to be a risky procedure in patients with invasive pulmonary aspergillosis, as* Aspergillus* invades tissue and removal of infected material can lead to massive hemorrhage [3]. However, bronchoscopy has recently been reported to be a safe procedure in children with leukemia and respiratory symptoms, where complications seemed rare and transient, but it should be noted that only 3 of the 31 patients in this report were infected with* Aspergillus* [8]. Bronchoscopy is the only method available for endobronchial disease assessment, and it also enables tracheal and bronchial tissue sampling for microscopic analysis and culture, allowing early diagnosis [2, 9]. Patients with AT present with nonspecific signs and symptoms and have radiographic studies that are often normal or without characteristic changes, requiring bronchoscopy for diagnosis [1, 2, 7, 10–12].

Several classifications of AT have been proposed based on bronchoscopic and microscopic appearances [13, 14], and it is likely that different forms represent a progressive spectrum of fungal invasion or that different forms of AT may coexist in the same patient [2]. In order to allow prediction of the clinical course and prognostication, a clinical classification as suggested by Krenke and Grabczak seems to be useful for clinicians [2]. Our patient fell into the second category of “highly immunocompromised patients with hematologic malignancies and/or patients undergoing hematopoietic cell transplantation,” which is described as having a much poorer outcome than lung transplant patients or in other groups of patients suffering from AT [2]. This is supported by several reports of patients with a combination of having undergone HCT with the diagnosis of* Aspergillus *tracheobronchitis who almost always had a fatal outcome [6, 12, 15–19]. The need for mechanical ventilation in immunosuppressed patients with AT also suggests a poor prognosis [7]. In pediatric patients with invasive aspergillosis, having undergone HCT led to a sixfold higher risk of death and the only predictor of improved survival was surgical intervention [20]. Survival of AT is possible if diagnosis is made promptly and provided the patient can tolerate removal of the infected material [5, 15, 16, 18, 21].

Overall, only very few cases of children with AT have been reported in the literature and therefore it is difficult to determine the prognosis of children affected by AT. Two recent case reports, with survival of a 5-year-old girl with Fanconi anemia with neutropenia [5] and a 6-year-old girl with acute lymphocytic leukemia after HCT with localized AT [4], are promising.

In our case, once the diagnosis of pseudomembranous and obstructive AT was made, management in the pediatric intensive care unit focused on deliberate ventilatory maneuvers, bronchodilation, minimizing airway turbulence, and dissolving the mechanical obstruction with nebulized acetylcysteine and amphotericin B in addition to mechanical removal of obstructive material. Our patient had already been treated with systemic antifungals for several days, broadened by addition of a second antifungal agent. Unfortunately, the infection had progressed too far to allow for her recovery.

In conclusion, the diagnosis of AT should be considered and bronchoscopy should be undertaken early in immunosuppressed pediatric patients with nonspecific respiratory symptoms and normal radiographic studies, to allow prompt diagnosis. Early diagnosis can enable timely initiation of therapy, including adequate systemic and local antifungal coverage, in addition to adjuvant local therapies and thorough removal of infectious material. Once respiratory failure occurs, the prognosis of AT remains poor.

## Abbreviations

AT: * Aspergillus* tracheobronchitis
CML: Chronic myelogenous leukemia
PICU: Pediatric Intensive Care Unit
CXR: Chest radiograph
HCT: Hematopoietic cell transplantation
GVH: Graft versus host disease.
